# Feature Selection for Predicting Tumor Metastases in Microarray Experiments using Paired Design

**Published:** 2007-03-20

**Authors:** Qihua Tan, Mads Thomassen, Torben A. Kruse

**Affiliations:** 1 Department of Biochemistry, Pharmacology and Genetics, Odense University Hospital, Odense, Denmark; 2 Department of epidemiology, Institute of Public Health, University of Southern Denmark, Odense, Denmark

**Keywords:** gene expression microarray, feature selection, metastasis, prediction

## Abstract

Among the major issues in gene expression profile classification, feature selection is an important and necessary step in achieving and creating good classification rules given the high dimensionality of microarray data. Although different feature selection methods have been reported, there has been no method specifically proposed for paired microarray experiments. In this paper, we introduce a simple procedure based on a modified t-statistic for feature selection to microarray experiments using the popular matched case-control design and apply to our recent study on tumor metastasis in a low-malignant group of breast cancer patients for selecting genes that best predict metastases. Gene or feature selection is optimized by thresholding in a leaving one-pair out cross-validation. Model comparison through empirical application has shown that our method manifests improved efficiency with high sensitivity and specificity.

## Introduction

Characterized by simultaneous profiling for the transcriptional activities of thousands of mRNA species in a human tissue, the DNA microarray technology represents an important high-throughput platform for analyzing and understanding human diseases. The tremendous potential provided by the new technology is serving us not only as a molecular tool for investigating disease mechanisms but also for classification and clinical outcome prediction (Dudda-Subramanya et al. 2003). Application of the technology in clinical oncology is demonstrating it as a powerful tool for refining diagnosis and improving prognostic prediction accuracy of cancer patients (Pusztai et al. 2003). Bioinformatics and biostatistics play important roles in such practices in establishing gene expression signatures or prognostic markers and in building up efficient classifiers (Asyali et al. 2006). Among the major issues in gene expression profile classification, feature selection is an important and necessary step in achieving and creating good classification rules given the high dimensionality of microarray data. There are various approaches for feature selection in the literature among which one common approach is the univariate selection scheme for selecting only genes with the highest statistical significance. Such an approach can be inadequate because (1) it tends to include elements that contribute highly redundant information and (2) it ignores the co-regulatory network in gene function. As a result, the univariate approach does not necessarily guarantee a best classifier (Ein-Dor et al. 2005; Baker and Kramer, 2006).

Tibshirani et al. (2002) proposed a Nearest Shrunken Centroids (NSC) method for both feature selection and tumor classification. In NSC, weak elements of the class centroids are shrunk or deleted via soft-thresholding to identify genes that best characterize each class. The method implemented in an R package (PAM, Prediction Analysis of Microarrays) performs well in identifying subsets of genes that can be used for classification and prediction. Although different feature selection methods have been reported for tumor classification (Inza et al. 2004), there has been no method specifically proposed for paired microarray experiments. In this paper, we introduce a simple feature selection procedure based on a modified t-statistic to microarray experiments using the popular matched case-control design and apply to our recent study on tumor metastasis in a low-malignant group of breast cancer patients for selecting genes that best predict metastases. Gene or feature selection is optimized by thresholding in a leaving one-pair out cross-validation procedure using the support vector machines (SVM) (Brown et al. 2000). Such an approach is necessary considering the advantages in a matched design because there are multiple factors (nodal status, tumor size, age, etc.) that convey important implications on tumor outcomes. Performance of the feature selection method is compared with that from PAM and from the ordinary paired t-test using receiver operating characteristics (ROC) analysis (Fawcett, 2006).

## Methods

Suppose in a paired microarray experiment, we have the gene expression values (usually in log scale) from *n* pairs of samples *j* = 1, 2, … *n*. For each gene *i* (*i* = 1, 2, … *p*), we obtain the differential gene expression in pair *j*, *d_ij_*, by substracting the expression value of the control from the case and calculate the mean difference as ${\bar{d}}_{i}=\sum_{j=1}^{n}d_{ij}/n$ and the standard error of *d¯_i_* as $s_{i}=\sqrt{\sum_{j=1}^{n}{\left(d_{ij}-{\bar{d}}_{i}\right)}^{2}/(n-1)}$ Now we can calculate the t-test statistic for the paired data as

(1) $$t_{i}=\frac{{\bar{d}}_{i}}{s_{i}}.$$

Similar to Tusher et al. (2001), we add a positive constant *s*_0_ to the denominator of (1) so that (1) becomes

(2) $${t^{\prime }}_{i}=\frac{{\bar{d}}_{i}}{s_{i}+s_{0}}=t_{i}\frac{1}{1+\frac{s_{0}}{s_{i}}}.$$

From (2) we can see that our modified t-statistic is a down-scaled t-statistic with the scaling determined by the ratio between *s*_0_ and *s_i_*. Once *s*_0_ is specified, the scaling has a large effect on genes with small standard errors. Following Tibshirani et al. (2002), we set *s*_0_ to the median value of *s_i_* (*i* = 1, 2, … *p*). For the purpose of feature selection, we specify a threshold Δ and pick up genes with $\left|{t^{\prime }}_{i}\right|-\Delta >0$. The optimal subset of genes is obtained through a leaving one-pair out cross-validation procedure using SVM. Similar to PAM, the optimal threshold Δ is determine through a grid search in which for each given Δ, the performance of classifier is judged by leaving one-pair out cross-validation to ensure that the training set and the prediction set are independent. The Δ that corresponds to the lowest classification error is taken as the optimal threshold. Once the optimal threshold Δ is determined, the overall optimal sub-set of genes is selected by applying the optimal Δ to the whole sample. The realization of SVM is done using the *svm* procedure in the R package e1071 (http://cran.at.r-project.org/src/contrib/PACKAGES).

In order to assess and compare our model performance with that from PAM and the ordinary paired t-test, we introduce the ROC analysis and calculate the area under an ROC curve (AUC). A ROC curve is a two-dimensional depiction of classifier performance which plots sensitivity on the *Y* and 1-specificity on the *X* axes. As such, a high-AUC classifier has better average performance than a low-AUC classifier (Fawcett, 2006) with AUC = 0.5 for a random classifier. ROC analysis is performed using the free R package *caTools*.

## Application

**Figure 1.**
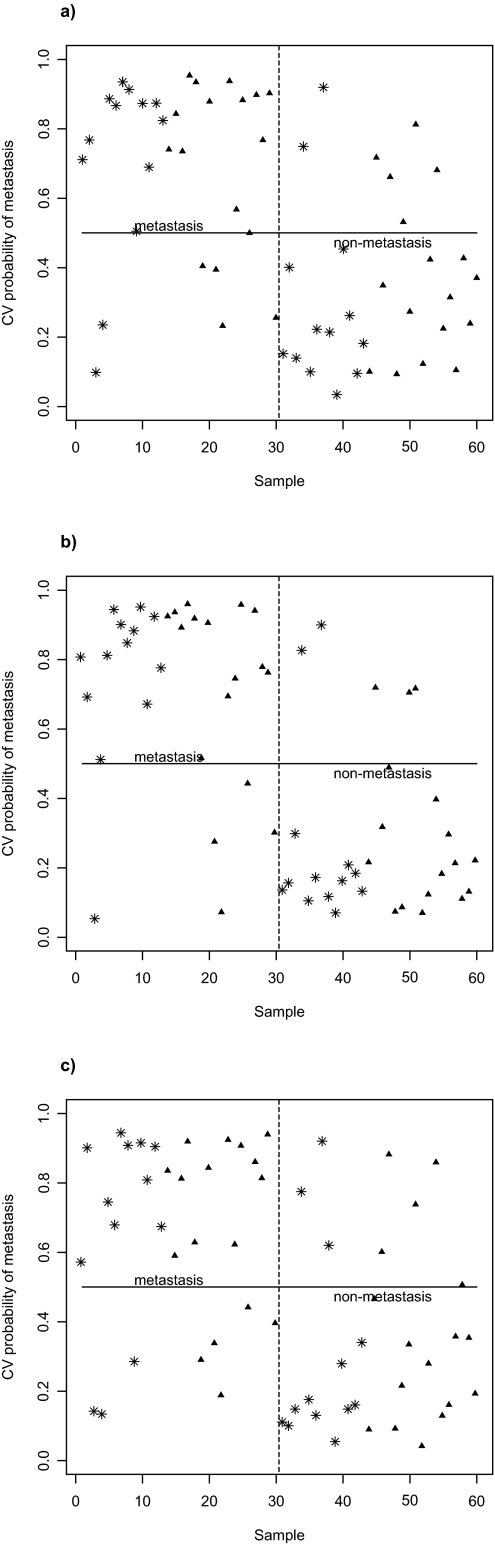
Probability of metastasis calculated by SVM using leaving one-pair out cross-validation based on the 32-gene signature by PAM (**1a**), the 5-gene signature by our new method (**1b**) and the 43-gene signature by paired t-test (**1c**) for the 13 pairs of low-malignant T1 (asterisk) and 17 pairs of low-malignant T2 (triangle) patients. The best performance is achieved by our 5-gene signature with improved prediction accuracy and better separation.

We apply our method to a microarray dataset on tumor metastasis from low-malignant breast cancer patients collected in our lab (Thomassen et al. 2006a). In this study, 13 low-malignant T1 (tumor size in diameter T ≤ 20 mm) and 17 low-malignant T2 (20 mm < T ≤ 50 mm) tumors from patients who developed metastases were matched to metastasis-free tumors from patients (followed up for about 12 years after diagnosis) of the same tumor type and according to year of surgery, tumor size, and age. Gene expression analysis was performed on 29K oligonucleotide arrays with duplicated measurements for each gene (Thomassen et al. 2006b). Data were normalized using the variance stabilization normalization method (Huber et al. 2002) implemented in the free R package *vsn* in Bioconductor (http://www.bioconductor.org). The study by Thomassen et al. (2006a) identified a 32-gene signature that classifies the 60 tumor samples with a mean accuracy of 78% (specificity 77%; sensitivity 80%) using leaving one-pair out cross-validation (Figure 1a). In the analysis, feature selection was done using the nearest shrunken centroids methods in the R package *pamr* (Tibshirani et al. 2002) and classification done using SVM in the R package *e1071*. Note that the feature selection procedure using *pamr* does not take the paired matching into account in identifying the subset of genes for training and prediction.

Using our method described above, we re-analyze the data by introducing the modified t-statistic for paired data in defining the gene expression signature for predicting metastases. Our analysis achieved an overall accuracy of 83% (Δ = 0.396) with a specificity of 83% and a sensitivity of 83% using a subset of only 5 genes (Figure 1b). Comparing Figure 1a with 1b, one can see that our method has improved separation based on prediction probability and increased efficiency (median of correct prediction probability: 0.88 versus 0.86 for metastasis and 0.84 versus 0.81 for non-metastasis). Interestingly, all the 5 selected genes are within the 32-gene list identified by PAM in Thomassen et al. (2006a). To further compare our analysis, we additionally introduce the ordinary paired t-test for gene selection. Here the thresholding is imposed upon the ordinary paired t-statistic, i.e. we pick up genes with | *t_i_* | −Δ > 0. Likewise, we again select the optimal subset of genes through cross-validation by leaving one-pair out. The classifier based on the expression signature specified by the ordinary paired t-test yields an average accuracy of 74% (specificity 74%; sensitivity 74%) when Δ is set to 3.1 (43 genes selected). The cross-validation probabilities plotted in Figure 1c shows that the model based on ordinary paired t-test has the lowest efficiency (median of correct prediction probability: 0.85 for metastasis and 0.83 for non-metastasis) even though the method makes use of the paired design.

**Figure 2.**
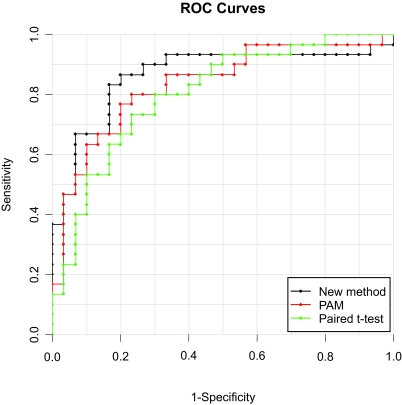
ROC analysis for model comparison with the dotted curves for the new method in black, for PAM in red and for the paired t-test in green. Since the black curve runs on top of the others in the upper-left triangle of the figure, our new method exhibits higher efficiency in its performance. The high AUC for our new method (0.86) indicates that it outperforms PAM (AUC = 0.83) and the paired t-test (AUC = 0.80).

We finally evaluate the overall performances of the 3 methods using ROC analysis. Based on the cross-validation probability of metastasis from SVM and the observed metastasis status for each sample, we are able to draw the ROC curves and show it in Figure 2 with the dotted curves for the new method in black, for PAM in red and for the paired t-test in green. Visualization of Figure 2 indicates that since the black curve runs on top of the other curves in the upper-left triangle of the figure, our new method exhibits higher efficiency as compared with the others. This is further confirmed by calculating the AUC, a standard summary metric for assessing the overall performance of a classifier. The high AUC for our new method (0.86) again shows that it outperforms PAM (AUC = 0.83) and the ordinary paired t-test (AUC = 0.80).

## Discussion

We have introduced a simple feature selection method for predicting tumor metastases in paired microarray experiments. Model comparison through empirical application has shown that our method manifests high efficiency and outperforms existing methods. As shown in the results section, the ordinary paired t-tests has the worst performance as compared with the other two methods which use modified t-statistics for thresholding to eliminate genes that do not contribute towards class prediction. Although both the modified and the ordinary paired t-statistics make use of the matched design, the better performance of our method is achieved by thresholding upon a new metric that is less dependent on gene-specific variances which helped to filter statistically significant genes due to small standard errors in their differential expressions. It is more interesting to compare the performances between our method and PAM. Although both methods use the modified versions of t-statistics, our method takes the following advantages of the paired design in selecting informative features. First, as a popular method in cancer research (Breslow and Day, 1990), the paired design helps to minimize the influence on tumor metastasis from non-transcriptomic factors such as age, clinical stage, treatment, etc (Gonzalez-Angulo et al. 2005). Second, in a transcriptomic study on tumor metastasis, these confounding factors not only affect the metastasis phenotype which is of our primary interest but could also influence the transcriptional profiles of genes. Ignoring these influences will simply introduce noise in feature selection resulting in low accuracy of the classifier.

A good classification signature should be a minimal subset of genes that is not only differentially expressed but also contains most relevant genes without redundancy (Peng et al. 2006; Baker and Kramer, 2006). A comparative analysis on data across several studies has found that classification rules for 5 genes can achieve comparable performance as that for 20 or 50 genes (Baker and Kramer, 2006). In our analysis, the high performance is achieved by basing our classifier coincidently on 5 informative genes. It is interesting that all 5 genes overlap with the 32-gene signature identified by PAM (Thomassen et al. 2006a) and 2 of the 5 genes overlap with the 70-gene signature from van’t Veer et al. (2002) in their studies on breast cancer metastases. Further information on the 5 selected genes is provided in Table 1.

Finally, it is necessary to point out that the paired experiment design in studying tumor metastasis using two-channel cDNA microarrays can be further advantaged by the reduced experimental cost when directly labeling, for example, metastasis mRNA with cy5 and non-metastasis mRNA with cy3 in each matched pair. Since our method works with the pair-wised difference in the log expression values, the feature selection algorithm is valid for both one- and two-channel microarray platforms. Overall, given the popularity of the pair matched design in cancer studies, we hope that our new method for feature selection can be of use in identifying efficient and informative gene expression signatures for predicting tumor metastases in clinical cancer research.

## Figures and Tables

**Table 1.** Information on the 5 selected genes.

**Gene symbol**	**GenBank accession**	**Description**	**Gene Ontology**
FLJ20354	NM_017779	Hypothetical protein FLJ20354, mRNA.	Intracellular signaling cascade
IMAGE:4081483	BC005998	Clone IMAGE:4081483, mRNA	Unknown
UBE2R2	NM_017811	Ubiquitin-conjugating enzyme E2R 2, mRNA.	Ligase activity; ubiquitin conjugating enzyme activity; Ubiquitin cycle; ubiquitin-ligase activity
ZNF533	NM_152520	Zinc finger protein 533	Unknown
DTL	NM_016448	Denticleless homolog	Unknown
